# Supplementary figures and images for: Metabolic flux from the Krebs cycle to glutamate transmission tunes a neural brake on seizure onset
Source: PLoS Genet. 2021 Oct 29;17(10):e1009871. doi: 10.1371/journal.pgen.1009871 (PMC8555787; doi:10.1371/journal.pgen.1009871)

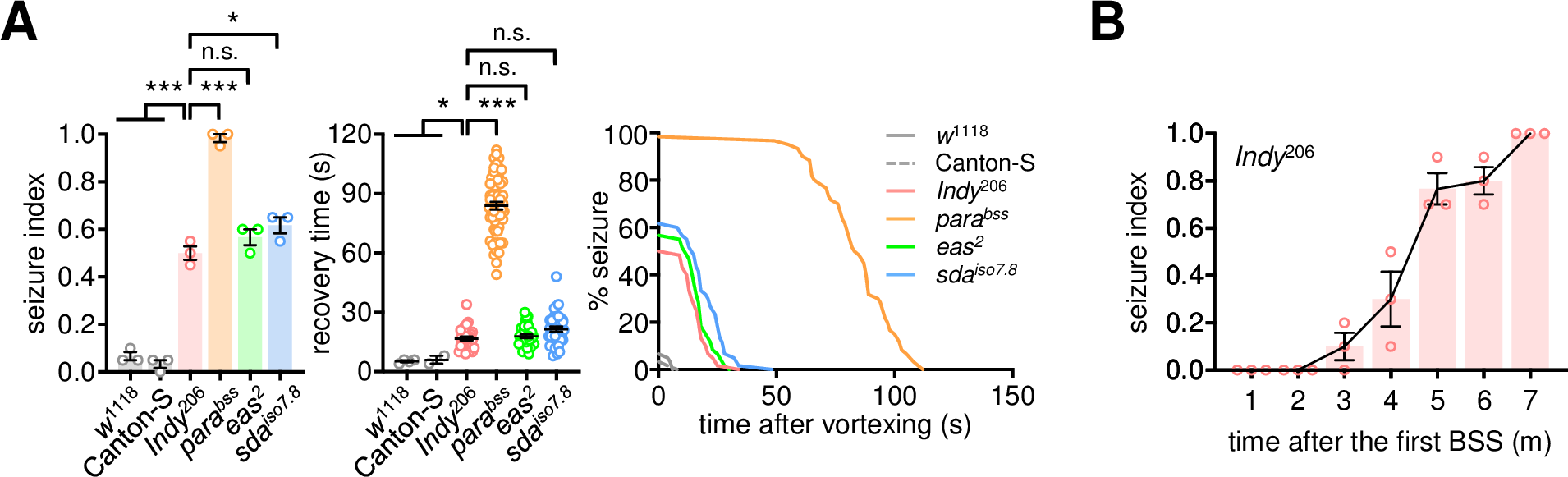

Supplement: S1 Fig — (A) The seizure phenotypes in Indy mutants were comparable to those observed in easily shocked (eas2) and slamdance (sdaiso7.8) but weaker than bang senseless (parabss) mutants. Quantitative analyses of BSS in individual flies were performed as described in Fig 1. Data represent means ± SEM (seizure index, n = 60 flies in 3 independent experiments; recovery time, n = 2–59 flies). n.s., not significant; *P < 0.05, ***P < 0.001, as determined by one-way ANOVA with Holm-Sidak’s multiple comparisons test. (B) Indy mutant seizure displays a refractory period after seizure recovery. The first mechanical stimulus was given to each fly and then the second mechanical stimulus was given only to BSS-positive animals at the indicated time after recovery from their first BSS. The seizure index was calculated in each experiment (n = 10 flies per time point) and averaged from three independent experiments. Error bars indicate SEM. (TIF) [file pgen.1009871.s001.tif]

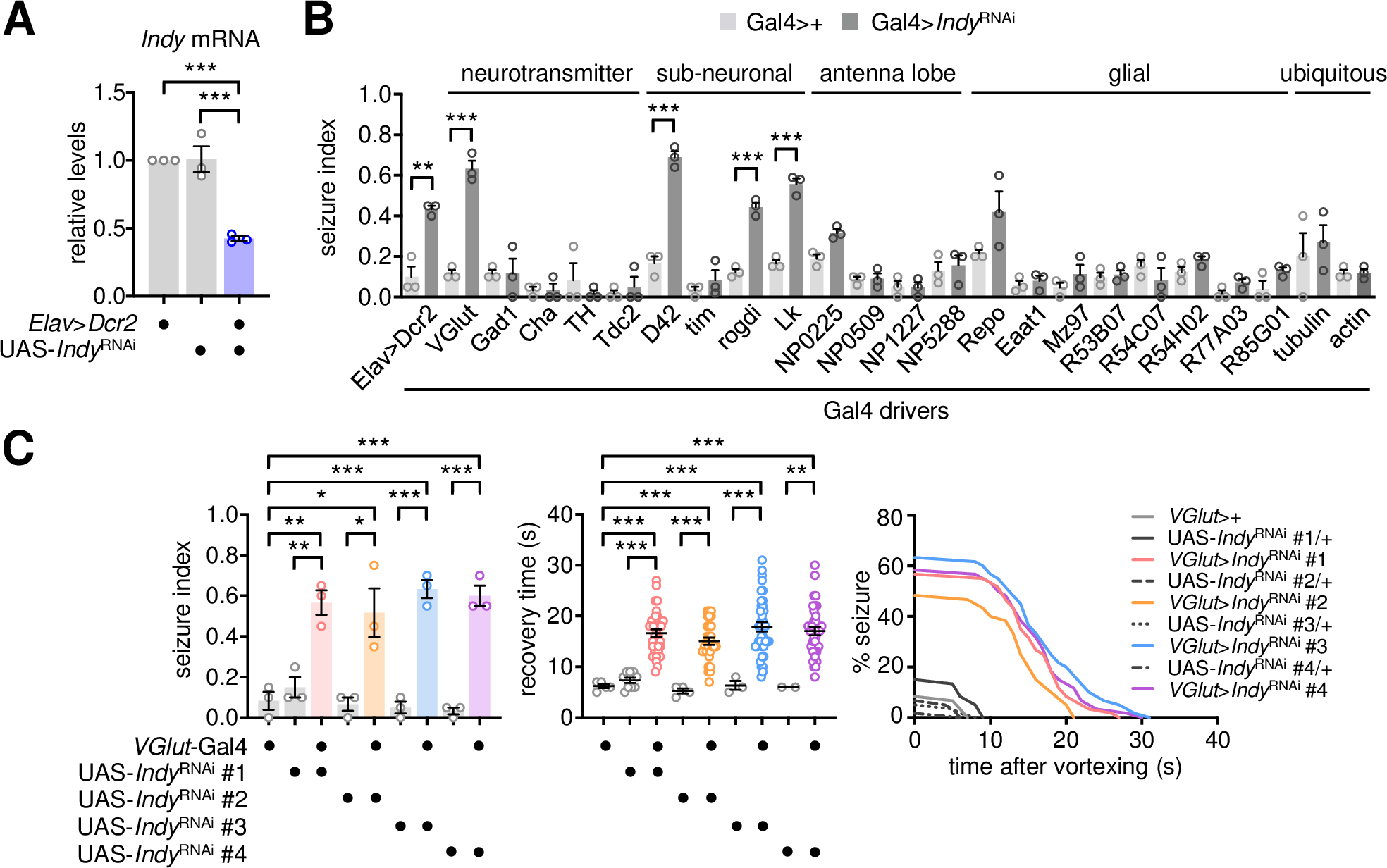

Supplement: S2 Fig — (A) Pan-neuronal overexpression of IndyRNAi transgene reduces endogenous Indy expression. Total RNA was purified from fly heads. Quantitative analyses of Indy and poly(A)-binding protein (normalizing control) mRNAs were performed using real-time PCR with gene-specific primer sets. The relative levels of Indy mRNA in each genetic background were calculated by normalizing to Elav>Dcr2 control (set as 1). Data represent means ± SEM (n = 3). ***P < 0.001 as determined by one-way ANOVA with Holm-Sidak’s multiple comparisons test. (B) A genetic screen identifies VGlut- and Lk-expressing neurons as neural loci important for Indy-dependent control of the seizure susceptibility. Quantitative analyses of BSS in individual flies were performed as described in Fig 1. Data represent means ± SEM (n = 60 flies in 3 independent experiments). **P < 0.01, ***P < 0.001, as determined by one-way ANOVA with Holm-Sidak’s multiple comparisons test. (C) The BSS induction by glutamatergic INDY depletion is consistently observed by independent Indy RNAi transgenes. Data represent means ± SEM (seizure index, n = 60 flies in 3 independent experiments; recovery time, n = 1–38 flies). *P < 0.05, **P < 0.01, ***P < 0.001, as determined by one-way ANOVA with Holm-Sidak’s multiple comparisons test. (TIF) [file pgen.1009871.s002.tif]

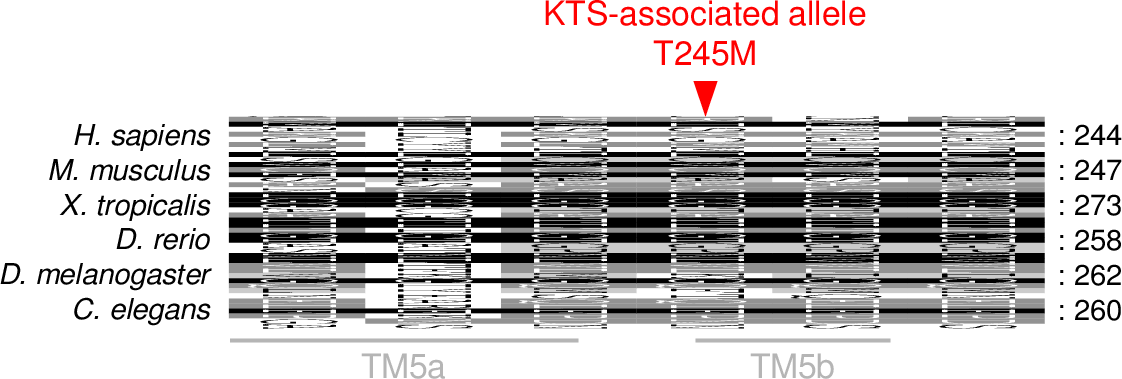

Supplement: S3 Fig — Drosophila IndyT245M mutant mimics the gene product of the KTS-associated 680C>T allele. TM, a transmembrane region. (TIF) [file pgen.1009871.s003.tif]

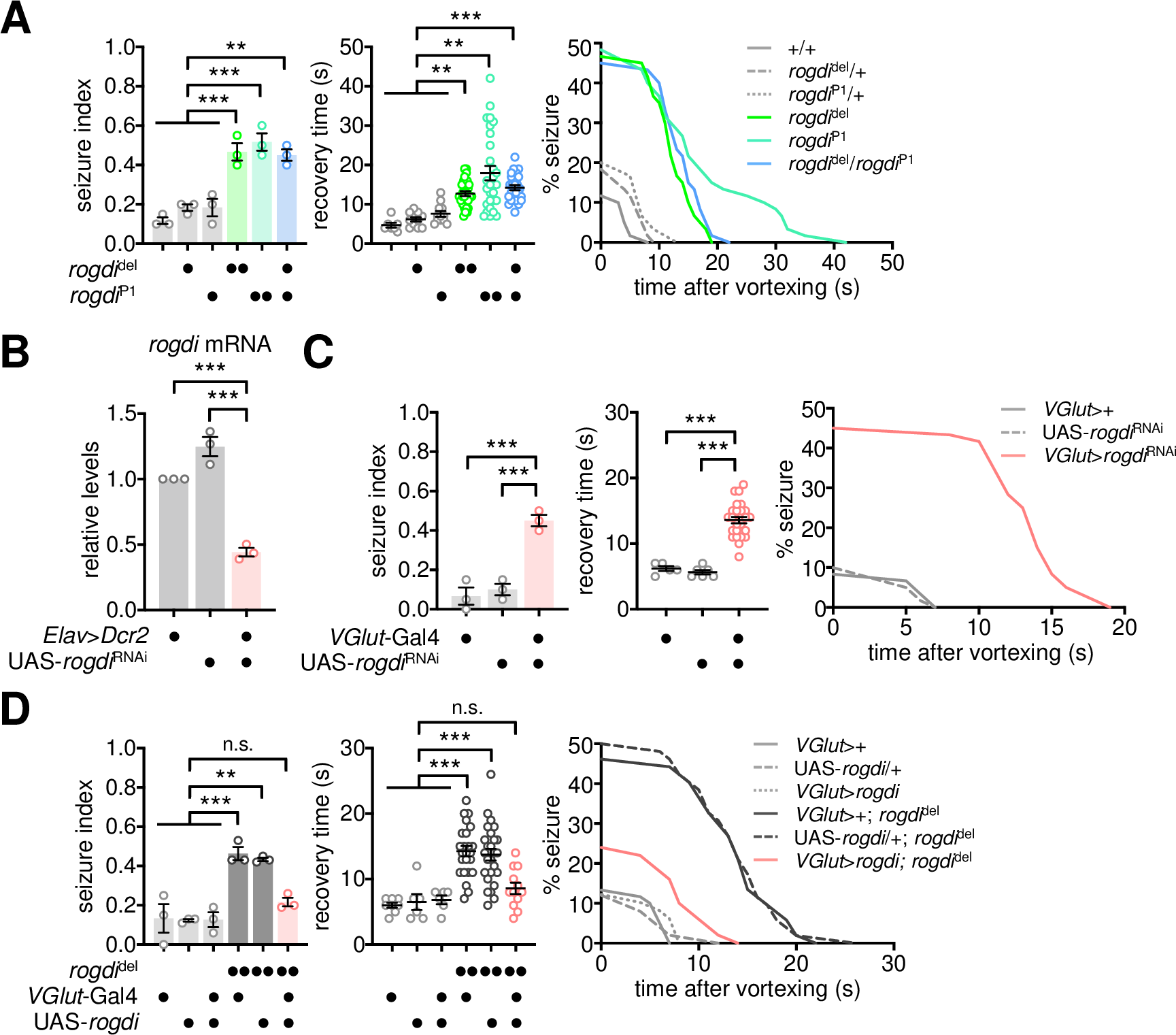

Supplement: S4 Fig — (A) rogdi mutants homozygous or trans-heterozygous for loss-of-function alleles display BSS. Quantitative analyses of BSS in individual flies were performed as described in Fig 1. Data represent means ± SEM. **P < 0.01, ***P < 0.001 as determined by one-way ANOVA with Holm-Sidak’s multiple comparisons test (seizure index, n = 60 flies in 3 independent experiments) or by Aligned ranks transformation ANOVA with Wilcoxon rank sum test (recovery time, n = 7–29 flies). (B) Pan-neuronal overexpression of rogdiRNAi transgene reduces endogenous rogdi expression in fly heads. Quantitative transcript analyses were performed as described in S2A Fig. Data represent means ± SEM (n = 3). ***P < 0.001 as determined by one-way ANOVA with Holm-Sidak’s multiple comparisons test. (C) ROGDI depletion in glutamatergic neurons is sufficient to induce BSS. Quantitative analyses of BSS in individual flies were performed as described in Fig 1. Data represent means ± SEM (seizure index, n = 60 flies in 3 independent experiments; recovery time, n = 5–27 flies). ***P < 0.001, as determined by one-way ANOVA with Holm-Sidak’s multiple comparisons test. (D) Transgenic overexpression of wild-type ROGDI in glutamatergic neurons rescues BSS in rogdi mutants. Data represent means ± SEM (seizure index, n = 49–60 flies in 3 independent experiments; recovery time, n = 6–26 flies). n.s., not significant; **P < 0.01, ***P < 0.001, as determined by two-way ANOVA with Holm-Sidak’s multiple comparisons test. (TIF) [file pgen.1009871.s004.tif]

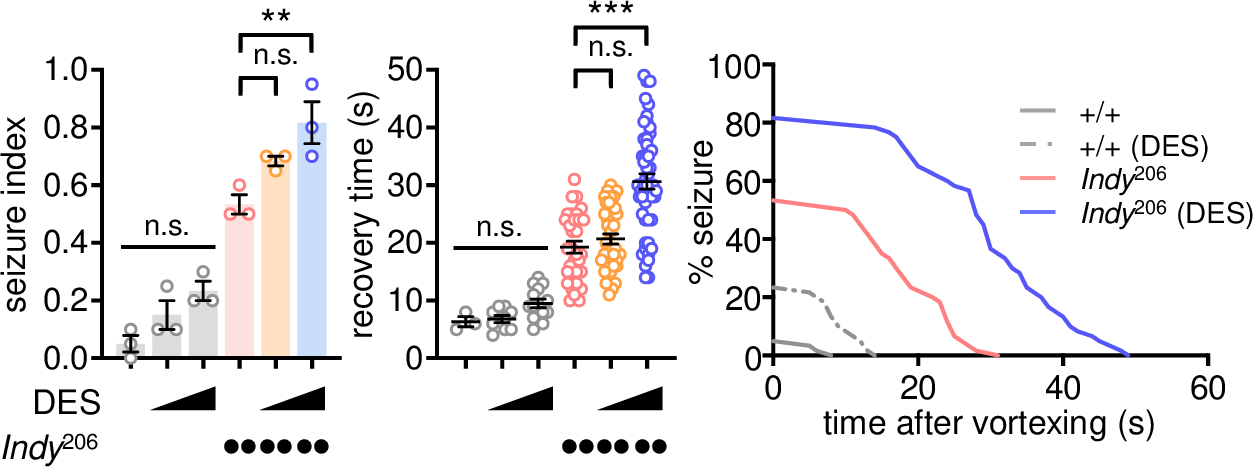

Supplement: S5 Fig — Flies were fed control or DES-containing food (10 or 20 μg/mg) for 3 d before the BSS assessment. Quantitative analyses of BSS in individual flies were performed as described in Fig 1. Two-way ANOVA detected a significant Indy x DES interaction effect on recovery time (P = 0.0421). Data represent means ± SEM (seizure index, n = 60 flies in 3 independent experiments; recovery time, n = 3–49 flies). n.s., not significant; **P < 0.01, ***P < 0.001, as determined by Holm-Sidak’s multiple comparisons test. (TIF) [file pgen.1009871.s005.tif]

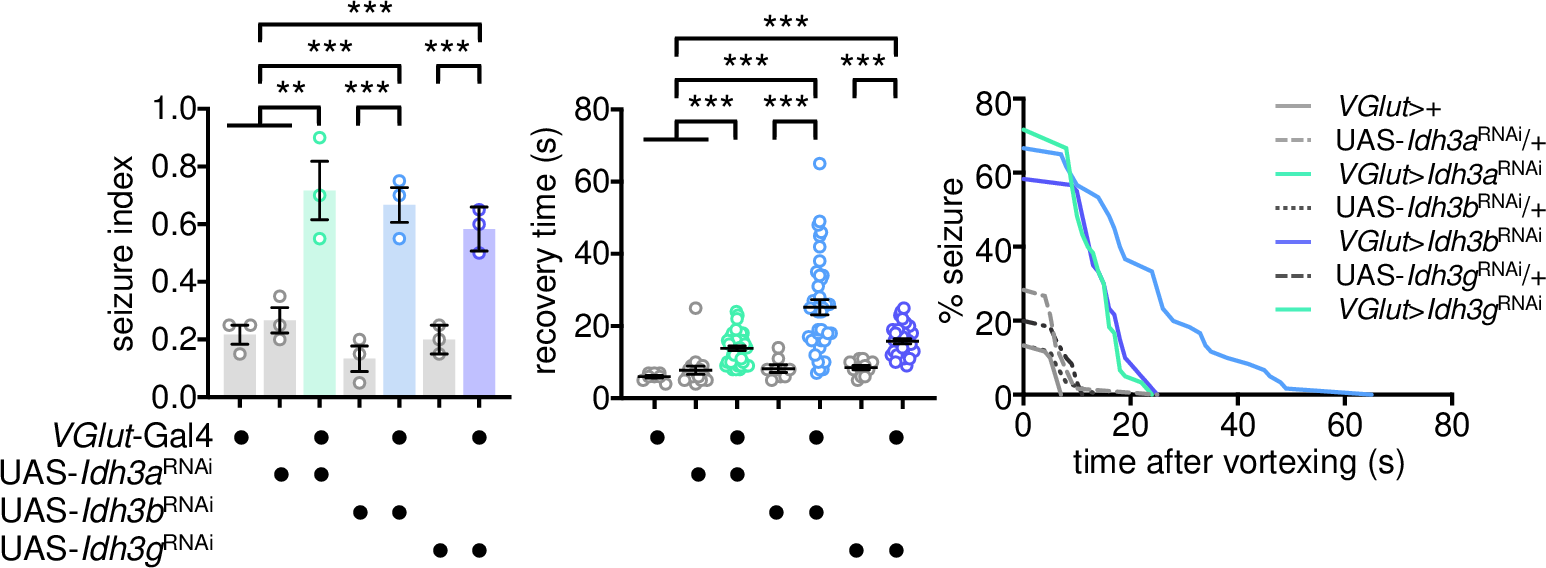

Supplement: S6 Fig — Quantitative analyses of BSS in individual flies were performed as described in Fig 1. Data represent means ± SEM. **P < 0.01, ***P < 0.001 as determined by one-way ANOVA with Holm-Sidak’s multiple comparisons test (seizure index, n = 60 flies in 3 independent experiments), by Aligned ranks transformation ANOVA with Wilcoxon rank sum test (recovery time, n = 8–43 flies for Idh3aRNAi or Idh3bRNAi), or by Welch’s ANOVA with Dunnett’s T3 multiple comparisons test (recovery time, n = 8–35 flies for Idh3gRNAi). (TIF) [file pgen.1009871.s006.tif]

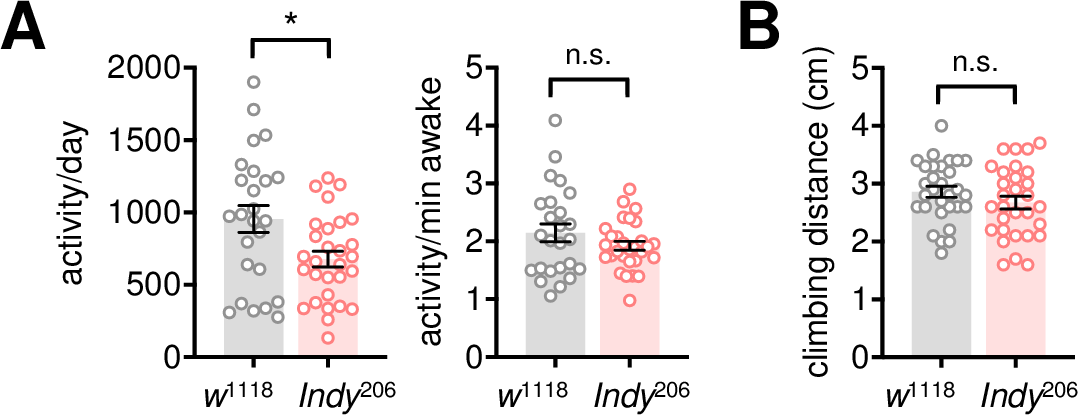

Supplement: S7 Fig — (A) w1118 control and Indy mutant flies show similar waking activities under 12-h light: 12-h dark (LD) cycles. Individual male flies were transferred to 65 × 5 mm glass tubes containing 5% sucrose and 2% agar food and entrained in LD cycles. Locomotor activities were indirectly measured by infrared beam crosses per minute using the Drosophila Activity Monitor system. Daily locomotor activity (activity/day) and waking activity (activity/min awake) were calculated in each fly on the fourth LD cycles and averaged (n = 25 and 29 flies for w1118 control and Indy206 mutants, respectively). Error bars indicate SEM. n.s., not significant; *P < 0.05 as determined by Mann-Whitney U test. (B) w1118 control and Indy mutant flies display comparable climbing activities. A group of 10 male flies was kept in the climbing chamber and then allowed to climb for 5 seconds after gentle tapping-down. Climbing distance in each fly was measured as the highest position during the recording and averaged (n = 30). Error bars indicate SEM. n.s., not significant as determined by Student’s t-test. (TIF) [file pgen.1009871.s007.tif]

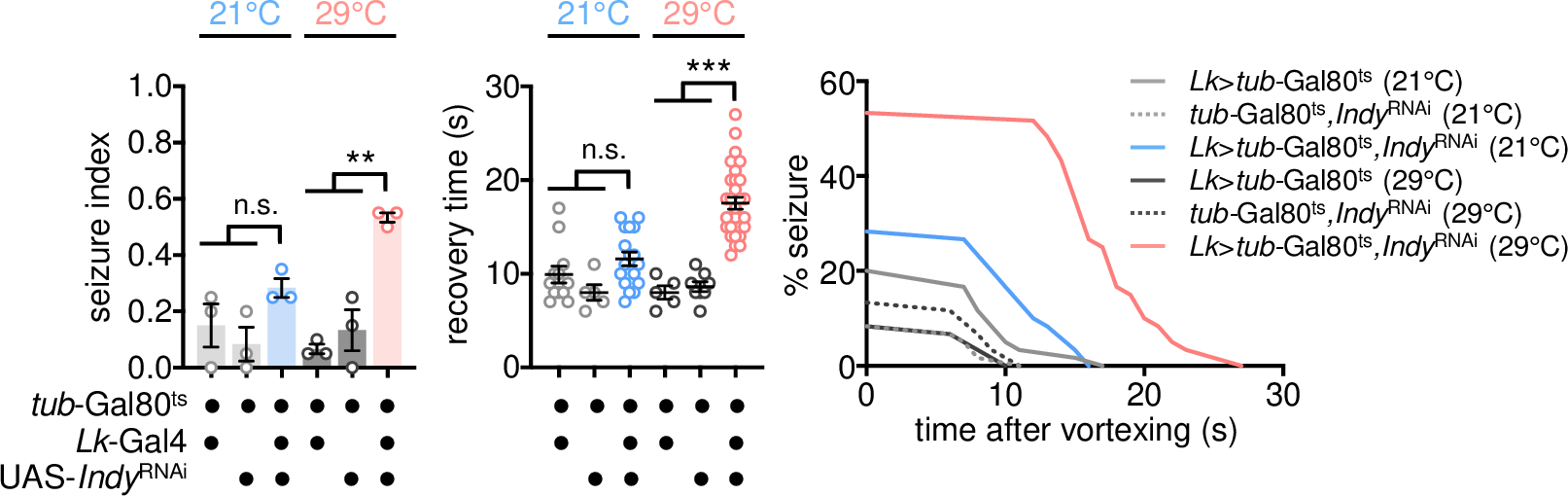

Supplement: S8 Fig — Adult-specific INDY depletion in LK neurons is sufficient to induce BSS. Transgenic flies were crossed and kept at 21°C to block the expression of IndyRNAi transgene using tub-Gal80ts. Adult flies were then incubated at 21°C (no depletion) or 29°C (RNAi-mediated depletion) for >24 hours prior to the assessment of BSS at the same temperature. Quantitative analyses of BSS in individual flies were performed as described in Fig 1. Data represent means ± SEM (seizure index, n = 60 flies in 3 independent experiments; recovery time, n = 5–28). n.s., not significant; **P < 0.01, ***P < 0.001, as determined by one-way ANOVA with Holm-Sidak’s multiple comparisons test. (TIF) [file pgen.1009871.s008.tif]

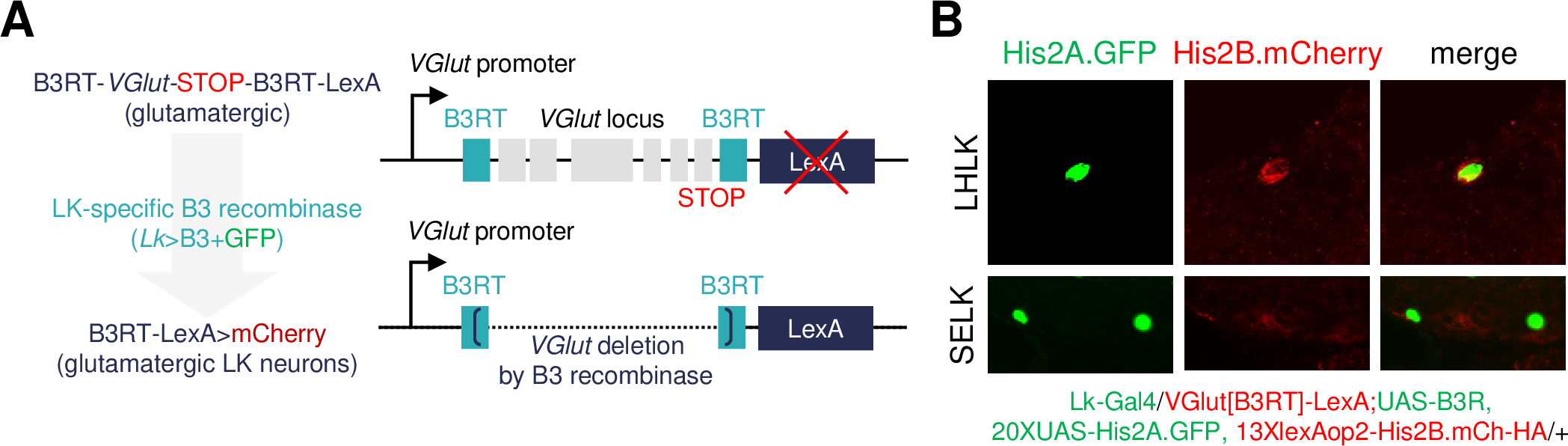

Supplement: S9 Fig — (A) A transgenic strategy for visualizing VGlut-expressing LK neurons by the fluorescent reporter proteins. The CRISPR-edited VGlut locus includes two B3 recombination target (B3RT) sites upstream of the LexA-coding sequence. LK neuron-specific overexpression of the B3 recombinase leads to the genomic excision, thereby allowing LexA expression only in VGlut-expressing LK neurons. LexA expression could be indirectly visualized by the transgenic mCherry reporter. (B) LHLK neurons, but not SELK neurons, express B3RT-LexA transgene from the VGlut-deleted locus. Representative confocal images of LHLK and SELK neurons in the adult fly brain were shown along with the full genotype. (TIF) [file pgen.1009871.s009.tif]

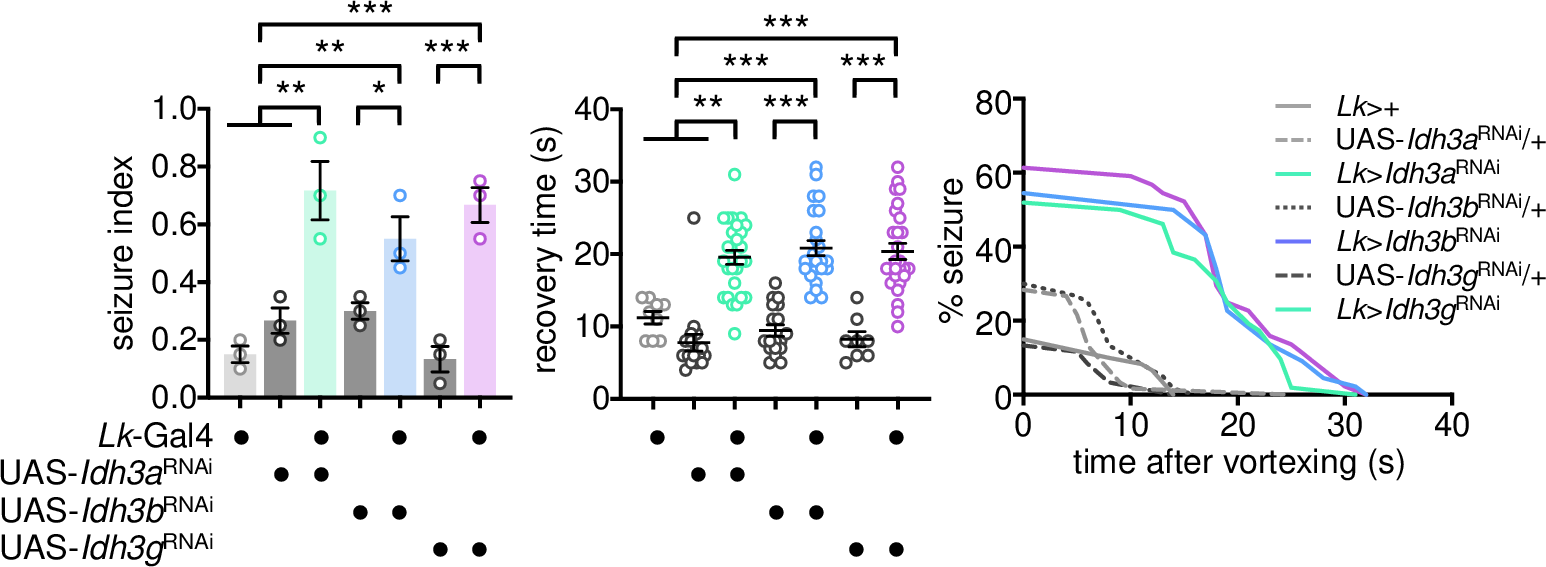

Supplement: S10 Fig — Quantitative analyses of BSS in individual flies were performed as described in Fig 1. Data represent means ± SEM (seizure index, n = 44–60 in 3 independent experiments; recovery time, n = 8–27). *P < 0.05, **P < 0.01, ***P < 0.001, as determined by one-way ANOVA with Holm-Sidak’s multiple comparisons test (seizure index), by Aligned ranks transformation ANOVA with Wilcoxon rank sum test (recovery time, Idh3a and Idh3b RNAi), or by Welch’s ANOVA with Dunnett’s multiple comparisons test (recovery time, Idh3g RNAi). (TIF) [file pgen.1009871.s010.tif]

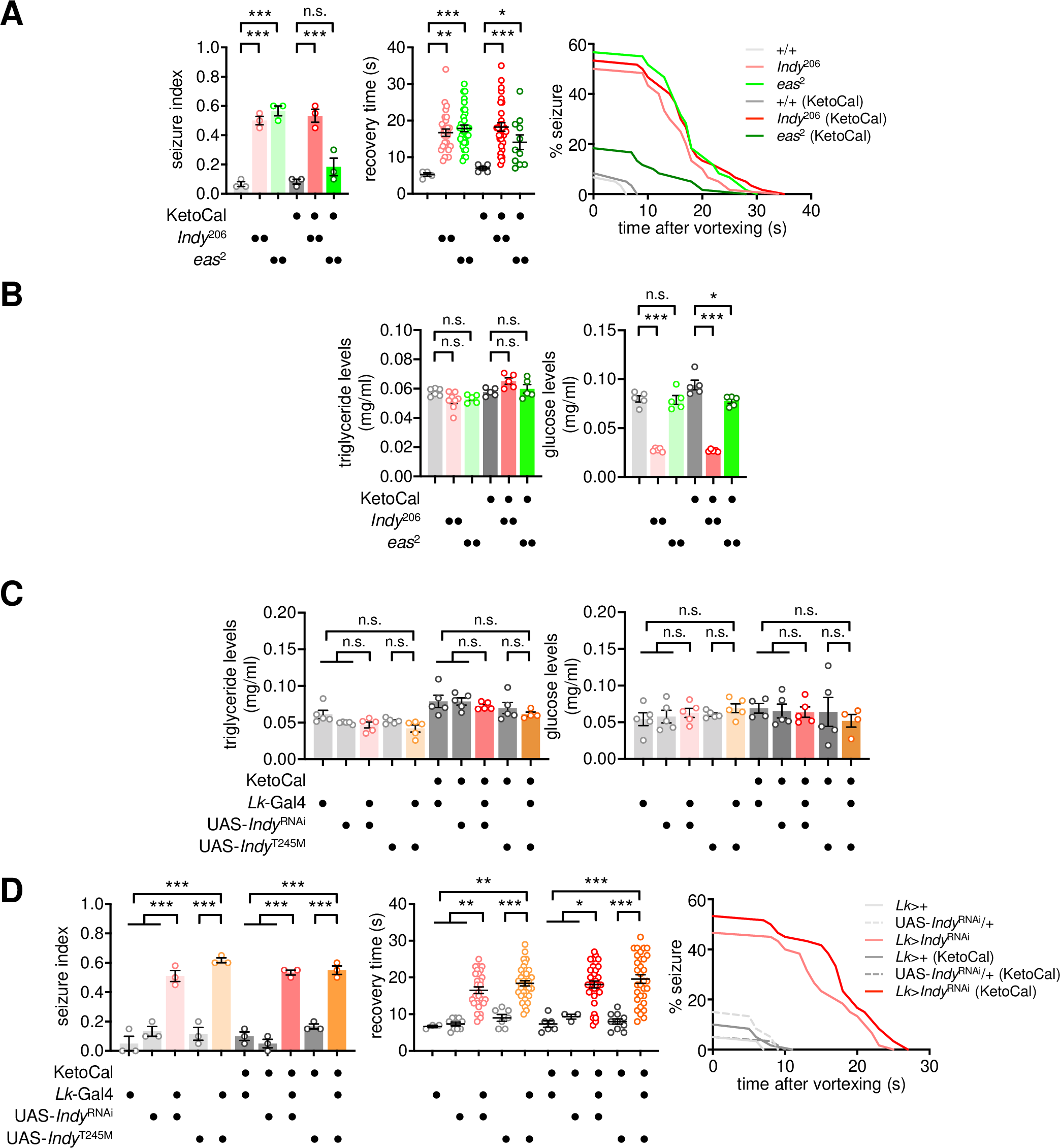

Supplement: S11 Fig — (A) A lipid-rich diet rescues BSS in eas2 mutants but not in Indy206 mutants. Flies were fed control or 5% KetoCal food for 3 d before the BSS assessment. Quantitative analyses of BSS in individual flies were performed as described in Fig 1. Two-way ANOVA detected a significant eas x KetoCal interaction effect on seizure index (P = 0.0006). Data represent means ± SEM (seizure index, n = 60 flies in 3 independent experiments; recovery time, n = 4–34). n.s., not significant; *P < 0.05, **P < 0.01, ***P < 0.001, as determined by Holm-Sidak’s multiple comparisons test. (B) The lipid-rich diet does not rescue low glucose levels in Indy mutants. Flies were fed control or 5% KetoCal (4: 1 = fat: carbohydrate plus protein) food for 3 d before harvesting. Triglyceride and glucose levels in whole-body extracts were quantified using standard curves. Two-way ANOVA detected significant Indy x KetoCal interaction effects on triglyceride (P = 0.0019) and glucose levels (P = 0.0228). Data represent means ± SEM (n = 5–8). n.s., not significant; *P < 0.05, ***P < 0.001, as determined by Holm-Sidak’s multiple comparisons test. (C) Loss of Indy function in LK neurons does not cause a change in metabolite levels. Data represent means ± SEM (n = 4–5). n.s., not significant as determined by two-way ANOVA with Holm-Sidak’s multiple comparisons test. (D) The lipid-rich diet does not rescue BSS induced by LK neuron-specific loss of Indy function. Two-way ANOVA detected no significant Indy x KetoCal interaction effect on seizure index (P = 0.7802 for IndyRNAi; P = 0.1725 for IndyT245M) and recovery time (P = 0.9534 for IndyRNAi; P = 0.7273 for IndyT245M). n.s., not significant; *P < 0.05, **P < 0.01, ***P < 0.001, as determined by Holm-Sidak’s multiple comparisons test. (TIF) [file pgen.1009871.s011.tif]

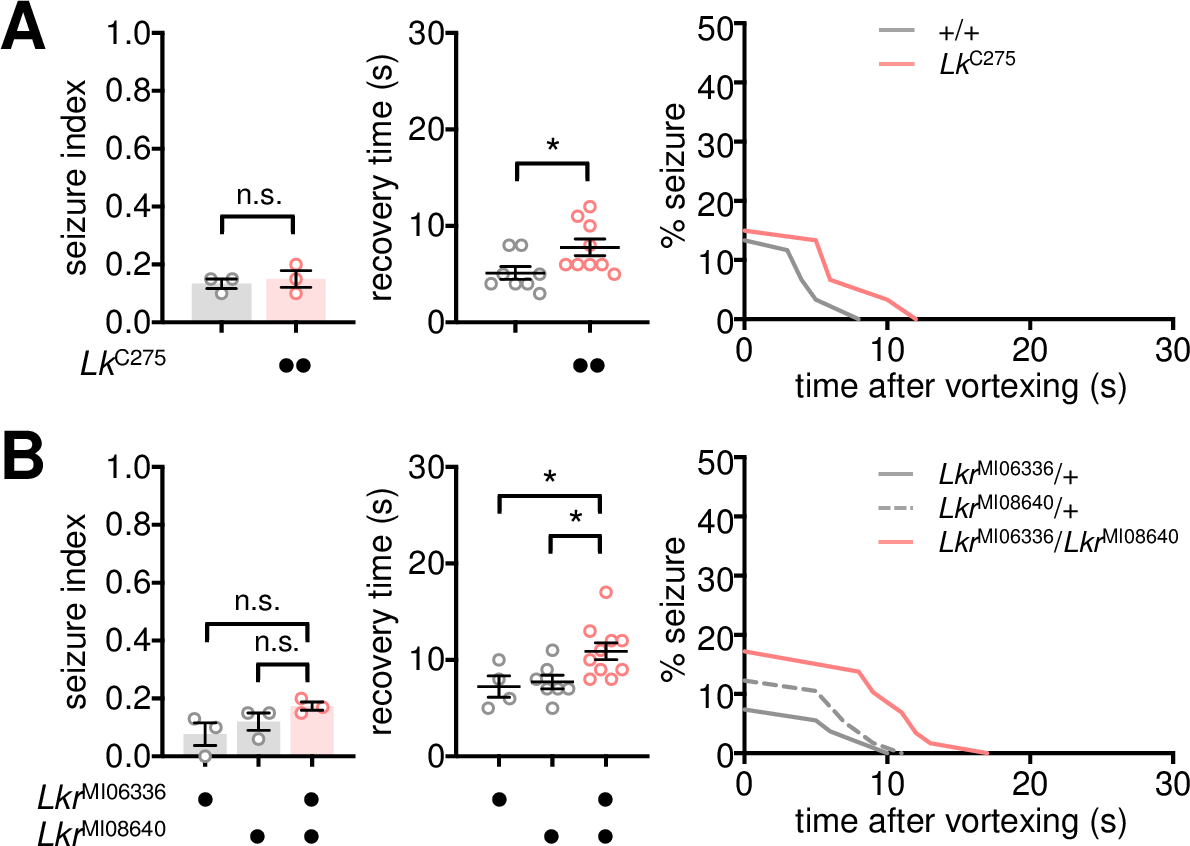

Supplement: S12 Fig — Quantitative analyses of BSS in individual flies were performed as described in Fig 1. Data represent means ± SEM. n.s., not significant; *P < 0.05 as determined by Mann-Whitney U test (seizure index, n = 60 in 3 independent experiments for Lk; recovery time, n = 8–9 flies for Lk) or by one-way ANOVA with Holm-Sidak’s multiple comparisons test (seizure index, n = 54–58 in 3 independent experiments for Lkr; recovery time, n = 4–10 flies for Lkr). (TIF) [file pgen.1009871.s012.tif]

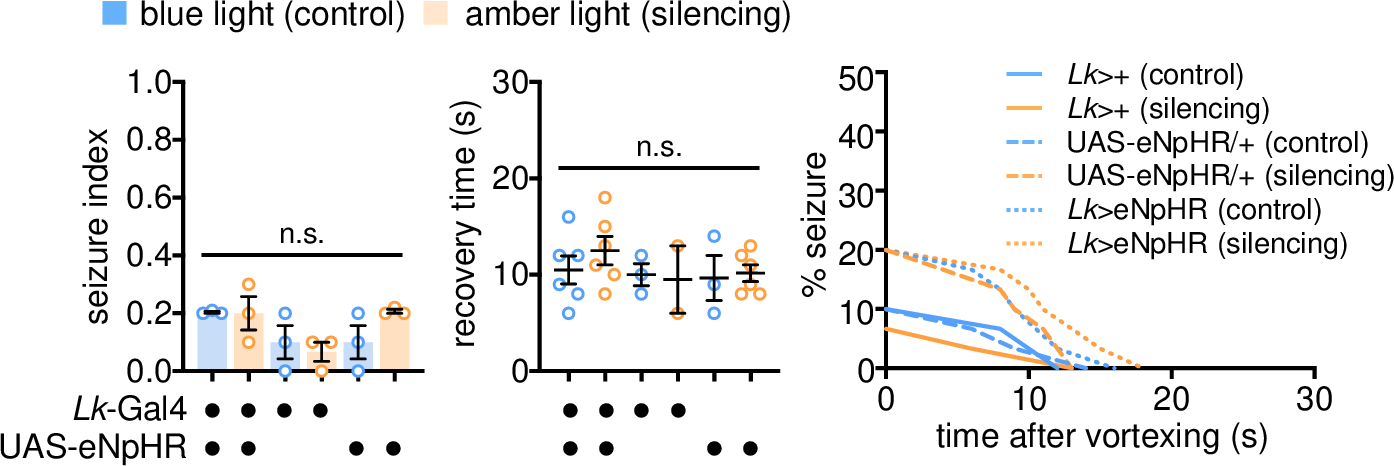

Supplement: S13 Fig — Transgenic flies were crossed and kept in constant dark. Any behavioral changes upon exposure to blue (no silencing) or amber light (silencing by eNpHR) condition were examined accordingly. Data represent means ± SEM (seizure index, n = 30 flies in 3 independent experiments; recovery time, n = 2–6 flies). No significant differences in seizure index and recovery time were detected by two-way ANOVA with Holm-Sidak’s multiple comparisons test. (TIF) [file pgen.1009871.s013.tif]
