# Supplementary material for: Metabolic flux from the Krebs cycle to glutamate transmission tunes a neural brake on seizure onset
Source: PLoS Genet. 2021 Oct 29;17(10):e1009871. doi: 10.1371/journal.pgen.1009871 (PMC8555787; doi:10.1371/journal.pgen.1009871)
Supplement: Attachment — Submitted filename: ResponseToReviewerComments - JeongLim2021.docx [file pgen.1009871.s016.docx]

Please find our point-by-point responses below in blue.

Reviewer #1: The Drosophila Indy gene encodes a fly homolog of the mammalian SLC13A5 citrate transporter. In this study, Jeong et al. found that loss-of-function Indy mutants displayed seizure-like behaviors in response to strong mechanical disturbances. Based on the experimental results obtained using genetic, optogenetic, and pharmacological approaches, the authors concluded that this behavioral abnormality of Indy mutants are caused by defective glutamate synthesis and glutamatergic transmission in the lateral horn leucokinin (LHLK) neurons, innervating the fan-shaped body. This is a carefully done study and findings are novel and interesting. In particular, it is surprising that, although Indy is expressed widely, seizure behaviors of the mutants are attributable to reduced Indy function in a specific pair of glutamatergic neurons in the brain (LHLK neurons). Several points need clarifying and certain statements require further justification.

1. A number of Drosophila “bang-sensitive” mutants (i.e., mutants with increased susceptibility to seizures following mechanical stimulation) have been identified. Some of them (e.g., easily shocked, bang senseless, and slamdance) have been extensively studied. Are the “bang-induced” seizure-like behaviors observed in Indy mutants similar to or different from the known bang-sensitive phenotypes? This is the first time that Indy mutants’ bang-induced phenotype is described. Please describe the phenotype in more detail in the context of other bang-sensitive phenotypes. It would be useful if a video clip showing the representative phenotype would be included in the supplemental information or a schematic figure describing the behavioral sequence could be shown (e.g., Fig. 1 in Howlett et al. (2013) for para[bss1]).

In the revised manuscript, we showed that *Indy* mutants display seizure-like behaviors comparable to those observed in *easily shocked* (*eas*) or *slamdance* (*sda*) mutants under our experimental conditions (S1A Fig). We also included a video clip representing the *Indy* mutant seizure in the supplementary information (S1 Movie).

2. Unlike mammals, Drosophila uses glutamate, instead of acetylcholine, as an excitatory neurotransmitter for motor neurons. The authors claim that reduced Indy function in glutamatergic neurons leads to suppression of glutamatergic transmission. It is expected that motor neurons in Indy mutants are suboptimal and that the mutants display defects in motor neuron physiology and motor behavior. Please refer to relevant previous studies, if any, that are consistent with the proposed effects of Indy loss-of-function on glutamatergic neurons.

It has been shown that *Indy* deficiency does not compromise physical activity (<https://pubmed.ncbi.nlm.nih.gov/11118146/>; <https://pubmed.ncbi.nlm.nih.gov/12626742/>; <https://pubmed.ncbi.nlm.nih.gov/15919590/>; <https://pubmed.ncbi.nlm.nih.gov/16891076/>; <https://pubmed.ncbi.nlm.nih.gov/19470468/>). We also confirmed it by comparing general locomotor activities (i.e., waking activity and climbing activity) in wild-type vs. *Indy* mutants (S7 Fig). It is thus likely that low glutamate levels/transmission in *Indy* mutants are limiting for glutamatergic seizure suppression in the context of a specific neural circuitry, but not for general motor function under non-stressful conditions. We revised our text accordingly.

3. In Indy mutants and VGlu-Gal4 mediated Indy knockdown flies, motor neurons are defective due to suppression of glutamatergic neurotransmission. In contrast, motor neurons are fully functional in the flies with Lk-Gal4 mediated Indy knockdown. When the bang sensitivity is increased due to reduced Indy function in LHLK neurons, I expect that the latter flies (Lk-Gal4 mediated Indy knockdown) display more severe seizures compared to the former flies (Indy mutants and VGlu-Gal4 mediated Indy knockdown flies) because of the difference in motor activity. However, the bang-sensitive seizure-like phenotypes are similar in these flies (Figs. 1B, 2B, and 5A). Any explanation?

We did not observe any gross motor defects in *Indy* mutants (S7 Fig). Please see our response to reviewer #1, comment #2 above.

4. Various Gal4 lines, including NP and R-lines, were used in a Gal4 mini screen (S1 Fig). Please indicate their specificity (tissue or cell type), if known, in the supporting information.

The specificity information for individual Gal4 lines was included in the revised manuscript (S2B Fig).

5. Knockdown of Indy in glutamatergic neurons leads to an increased sensitivity to bang-induced seizures. How specific is this behavioral phenotype to Indy knockdown in glutamatergic neurons? Indy knockdown in cholinergic, GABAergic, or serotonergic neurons does not have any effect? Do the NP- and R-lines used in this study include Gal4 lines driving Indy-RNAi expression in neuronal subsets other than glutamatergic neurons?

In the revised manuscript, we tested additional Gal4 lines specific to individual neurotransmitters and validated the specificity of glutamatergic *Indy* phenotypes (S2 Fig). We also indicated the specificity information for Gal4 lines used in our original mini-screen (S2B Fig).

6. Page 2, 2nd paragraph: It’s not appropriate to call mouse SLC13A5 INDY.

We revised the text.

7. Page 4, the 1st sentence: “Glutamate and GABA are two major neurotransmitters that mediate transmission at excitatory and inhibitory synapses, respectively”. In flies, they are acetylcholine and GABA, respectively.

We revised the text.

8. S4B Fig: Describe how the experiment was done.

We visualized the cell-type-specific expression of *Lk*, *Indy*, and *VGlut* genes in the adult fly brain using the published scRNA-seq database (<http://scope.aertslab.org/>). The other reviewer, however, asked us to experimentally validate *VGlut* expression in LHLK neurons. We thus provided new evidence for the glutamatergic nature of LHLK neurons (S9 Fig) and removed the original S4B Fig from the revised manuscript. Please see our response to reviewer #2, major concern #1.

Reviewer #2: Overall recommendation: Accept with revisions

In this well-controlled and interesting manuscript, Jeong et al find that knockdown or knockout of Indy, the Drosophila homologue of a gene implicated in a human seizure disorder, causes a bang-sensitive seizure behavior in flies. They then outline a mechanism by which losing Indy function leads to decreased TCA cycle activity and associated glutamate production, which acts as the primary cellular mechanism underpinning the bang-sensitive seizure susceptibility. Screening for neurons that can recapitulate this global phenotype, they then discover a set of neurons that seem to be responsible for gating the Indy¬-associated bang-sensitive phenotype and further map aspects of a preliminary neural circuit for bang-sensitive seizures. This circuit mechanism is bolstered with both genetic knockouts/knockdowns and optogenetic manipulation. Together, then, this manuscript establishes two interesting findings: it uncovers a potential cellular mechanism for a disease-causing mutation in humans and outlines the beginnings of a novel neural circuit governing a bang-sensitive seizure phenotype in flies. The manuscript is clearly written and the experiments robust and well-controlled and will likely prove interesting to a wide audience.

Major Concerns

1) The authors use single cell data from another publication to demonstrate that there is evidence of glutamatergic signaling machinery in LK neurons. This data as presented seems to rely on a single cell in the database that expresses both LK and VGlut, which could be correctly identifying the studied neurons or could easily be due to statistical randomness. Given the fact that this claim goes against the assumptions of the field, it would be more convincing to provide staining, in situ hybridization, or tagged protein localization evidence of Vglut in these LK neurons. Additionally, more information on how the single-cell data was visualized and manipulated should be included in the methods section if this data is retained in the final manuscript.

To validate *VGlut* expression in LHLK neurons, we employed a genome-edited *VGlut* allele (B3RT-dVGlut-B3RT-LexA) (<https://pubmed.ncbi.nlm.nih.gov/32097408/>). In this intersectional strategy, Lk-Gal4 specific expression of the B3 recombinase leads to the genomic excision of *VGlut* coding sequence flanked by the B3 recombination target sites (B3RTs), thereby driving the downstream expression of a transgenic LexA driver only in *VGlut*-expressing LK neurons (S9A Fig). When we visualized the spatial expression of Lk-Gal4 and B3RT-dVGlut-B3RT-LexA with GFP and mCherry, respectively, LHLK neurons but not SELK neurons displayed the overlapping expression of the two reporter proteins (S9B Fig). As we provided this new experimental evidence for *VGlut* expression in LHLK neurons, we removed the single-cell data from our revised manuscript.

2) The manipulation of GDH appears to this Reviewer to be an especially critical experiment as it serves as a clear link between TCA metabolism and glutamate transmission (as opposed to other downstream effects of TCA metabolism such as cell health). Therefore, I would find it extra convincing for the phenotype demonstrated using epigallocatechin gallate feeding to be recapitulated with a temperature sensitive RNAi or similar genetic approach. Alternatively, if GDH overexpression suppressed the effect of the GDH inhibitor in a cell-specific fashion this would also add certainty to that result.

Constitutive GDH depletion in glutamatergic neurons caused seizure-like behaviors (data not shown) but we had some difficulties in optimizing the transgenic combinations of *Indy* and *Gdh* to genetically demonstrate their significant interactions. Nonetheless, we tested an additional GDH inhibitor (i.e., diethylstilbestrol) and detected its significant interaction with *Indy* mutation on the seizure phenotypes, excluding possible off-target effects (S5 Fig)

3) The assumption of many experiments involving VGlut overexpression appears to be that overexpressing VGlut will compensate for lower levels of glutamate by increasing synaptic loading. Because the authors already have the capacity for GluSnFr visualization as shown later in the manuscript, it would be compelling to show that VGlut overexpression leads to greater extracellular glutamate release in the Indy-RNAi background, as such an experiment would argue against alternative changes in glutamate/glutamine cycling induced by VGlut overexpression.

In the revised manuscript, we showed that *VGlut* overexpression restores the iGluSnFR signals in the Indy-RNAi background (Fig 5A), consistent with its behavioral rescue (Fig 5B).

4) The authors use a bang-sensitive protocol that is different from most other labs - they vortex for 25 second (long) versus 10 seconds (normal), and the "seizures" are much shorter--they observe ~20 seconds versus 1-2 minutes in bang-sensitive mutants. Is this really seizure? There is no electrophysiology to address that. In addition, the pathway they have chosen to explore and many of the manipulations would be expected to have serious consequences on metabolism (e.g. TCA cycle)...is it possible that rather than seizure this is an energy deficiency? Are there controls they could do to address this?

*Indy* mutant seizure was comparable to *eas* and *sda* mutants under our experimental conditions, and it was weaker than *para*[bss] (S1A Fig). To examine the correlation between seizure phenotypes and metabolic status in genetic *Indy* models, we employed a lipid-rich diet (i.e., 5% KetoCal 4:1 supplement) that had been shown to rescue seizure mutants (<https://pubmed.ncbi.nlm.nih.gov/31260938/>). Under our experimental conditions, the KetoCal supplement partially but significantly suppressed *eas* mutant seizure while negligibly affecting the seizure phenotypes in *Indy* mutants (S11A Fig). *Indy* mutants displayed low glucose levels under our experimental conditions (S11B Fig), consistent with their energy deficiency (<https://pubmed.ncbi.nlm.nih.gov/19470468/>). The KetoCal diet did not restore glucose levels in *Indy* mutants (S11B Fig). Nonetheless, loss of *Indy* function in LK neurons did not affect the baseline levels of triglyceride and glucose (S11C Fig). LK neuron-specific seizure phenotypes were also insensitive to the lipid-rich diet (S11D Fig), indicating that energy deficiency may not be necessarily responsible for *Indy*-relevant seizure. As the other reviewer also raised a similar concern (reviewer #3, comment #3), we appropriately acknowledged this issue in the revised manuscript and discussed several lines of our evidence that supports the mechanistic specificity of *Indy*-dependent seizure control. These include, but are not limited to, the rescue of *Indy*-relevant seizures by the overexpression of non-metabolic VGLUT or by the optogenetic excitation of LK neurons only during a mechanical stimulus.

Minor Concerns

1. The paper introduces both Indy and rogdi as Drosophila homologues of Kohlschütter-Tönz syndrome and mentions both in the introduction and discussion. However, while a small amount of compelling evidence is presented in the supplements that a similar mechanism links rogdi and indy loss-of-function to this seizure model, the vast majority of the mechanistic characterization of the manuscript focuses on indy and thus the extent of discussion of rogdi in the introduction appears somewhat speculative.

As the other reviewer raised a similar concern (reviewer #3, comment #8), we appropriately revised our text for *rogdi*.

2. The labels of Figure 2c are difficult to read. I fully understand the authors’ decision to only have one set of x-axis labels to save space, but the complexity of the x-axis labels makes it very difficult to understand the upper graph.

We revised the figure to improve its readability.

3. The authors refer to depleting INDY expression – only Indy transcripts can change in expression, INDY protein should change in abundance (if this is indeed what is indicated by the change in nomenclature here)

We corrected the text throughout the revised manuscript.

4. Several times the authors use “On the other hand” in the context of information that is logically consistent with the previous sentence, which confused me as this phrase traditionally introduces information that is contradictory to the previous sentiment in some way.

We corrected the text throughout the revised manuscript.

5. Removal of the word “the” before metabolic challenge in the introduction.

Removed.

6. I would like the praise the authors for not attempting to statistically analyze the curves of seizure behavior over time in addition to seizure index and recovery time as such analysis quickly get statistically dubious.

We agree with the reviewer.

Reviewer #3: In the manuscript titled “Metabolic flux from the Krebs cycle to glutamate transmission tunes a neural brake on seizure onset”, Jeong et al. show that mutations in the citrate transporter gene Indy can lead to bang-sensitive seizures in Drosophila. Based on extensive genetic data, they propose a model which suggest that Indy affects glutamate metabolism in a pair of glutamatergic neurons, which act as an excitatory break on the onset of more generalized seizures. This is a well-written manuscript, which provides diverse empirical evidence for the proposed model. However, several concerns about data robustness and experimental approach require additional clarifications. Below are comments that I hope the authors will find useful:

1. The mechanistic model proposed here is based on possibly interesting Drosophila data. However, its true relevance to the early-onset of seizures in KTS might be overstated. The authors should make it clear to the readers that in contrast to the mammalian brain, the primary excitatory neurotransmitter in the fly brain is acetylcholine, not glutamate**.** Instead, glutamate plays a key role as the primary excitatory neurotransmitter at the fly’s NMJ. Therefore, if the link between the TCA cycle and neuronal glutamate metabolism is conserved across the fly and human nervous systems then one might expect the effects of Indy mutations to lead to higher seizure threshold relative to wild type animals.

In the revised manuscript, we clarified the difference in the primary excitatory neurotransmitters between *Drosophila* and mammals and toned down the relevance of our model to KTS-associated seizures. Low glutamate levels in *Indy* mutants may not be limiting for their general motor function given that *Indy* mutants displayed general motor function comparable to wild-type (S7 Fig). We reason that LK neurons are more sensitized to the link between the TCA cycle and glutamate metabolism for their “seizure-suppressing” glutamate transmission since this small group of neurons was necessary and sufficient for *Indy*-relevant seizure. Please also see our response to a relevant reviewer concern above (reviewer #1, comment #2)

2. Some of the data presented in the supplementary figures is confusing. For example, the authors use the pan-neuronal elav-GAL4 driver to show that the Indy RNAi transgene is effective in reducing Indy mRNA levels. Yet, the elav>Indy-RNAi F1 flies show no seizure hypersensitivity. It is puzzling why the authors did not also include the well-established GAL4 drivers for GABAergic and cholinergic neurons. Did the author include UAS-Dcr transgene in all their focal crosses?

In the original manuscript, we tested two independent Elav-Gal4 lines (i.e., “Elav>Dcr2”, X chromosome insertion with UAS-Dcr2 for qPCR validation; “Elav”, 2nd chromosome insertion without UAS-Dcr2 for Gal4 screen) but we did not include the UAS-Dcr2 transgene in our Gal4 screen. INDY depletion by the Elav>Dcr2 caused the seizure phenotypes, consistent with qPCR data. We reason that Elav-Gal4 expression from the second chromosome insertion may be weak particularly in the seizure-suppressing glutamatergic neurons, including LHLK neurons. To avoid any confusion, we revised the supplementary figure (S2B Fig). As the other reviewer also asked us to validate the specificity of INDY-depletion phenotypes in glutamatergic neurons (reviewer #1, comment #5), we tested additional Gal4 drivers and found that INDY depletion in GABAergic or cholinergic neurons does not cause a bang-sensitive seizure (S2B Fig).

3. Linking metabolism to hyperexcitability and other aspects of neuronal physiology is always difficult because demonstrating specificity of genetic and pharmacological manipulations on phenotype, especially when it is measured at the organismal level, is almost impossible. The authors should acknowledge this in their introduction and discussion, and should address the many possible mechanistic confounds related to their proposed interpretation of the data. For example, long-term knockdown of Indy in any cell type will lead to physiological stress, and poor phenotypic outcomes

In the revised manuscript, we appropriately acknowledged the reviewer concern above and discussed several lines of our evidence that supports the mechanistic specificity of *Indy*-dependent seizure control. These include, but are not limited to, the rescue of *Indy*-relevant seizures by the overexpression of non-metabolic VGLUT or by the optogenetic excitation of LK neurons only during a mechanical stimulus. Please also see our response to reviewer #2, major concern #4.

4. One major issue with the experimental design as described in the methods is that the authors may have not addressed some the known issues with strong effect of genetic background on bang-sensitivity. No specific information for most GAL4 lines used for the screen shown in Fig. S1, or what was the specific genetic background of control RNAi animals is not provided. Furthermore, the specific RNAi line used by the authors in their screen is pupal lethal when broadly expressed, and has been discarded by the NIG due to off-target effects, and no longer available.

We agree with the reviewer that genetic backgrounds could substantially affect any behavioral outputs. Accordingly, we revised the method to better describe our experimental design. *Indy* lines harboring each mutant allele (*Indy*^206^, *Indy*^302^) had been outcrossed six times to our genetic control (*w*^1118^, BL5905) for isogenization. All the behavioral tests for heterozygous, homozygous, and trans-heterozygous mutants were conducted with the isogenized *Indy* lines and *w*^1118^ control. For transgenic lines, we compared seizure behaviors in trans-heterozygous animals to those in all appropriate heterozygous controls. In most cases, we validated the transgenic phenotypes using additional transgenic combinations or independent transgenes. For example, in our original manuscript, *Indy* RNAi phenotypes were confirmed using *Indy*^T245M^ transgene that mimicked the KTS patient-specific allele. We further employed three additional *Indy* RNAi lines (i.e., UAS-*Indy*^RNAi^ #2, #3, and #4) in the revised manuscript to confirm the seizure phenotypes by INDY depletion (S2C Fig). The specificity information for individual Gal4 lines was also included in the revised manuscript (S2B Fig).

5. If five flies were used in each trial bang-sensitivity trial, the lowest % recovery possible at any given time point is 20% (1/5 flies). Therefore, it is puzzling how it is possible to have values below 20% in Fig. 1. Furthermore, since each recovering fly represents 20% of the population, it is confusing why the curves seem to describe continuous data.

Under our standard conditions, we tested 20 flies per genotype/condition in each experiment (i.e., 5 flies per group x 4 groups = 20 flies per genotype/condition). The seizure index was calculated by the ratio of the number of seizure-positive flies to the total number of flies tested in each experiment (e.g., 3/20 = 0.15) and averaged from three independent experiments (i.e., 3 data points in the figure). However, recovery time was measured from individual flies displaying seizure-like behaviors, and % seizure was calculated by the ratio of the number of seizure-positive flies to the total number of flies tested in all three experiments (e.g., 1/60 = 1.7%) at each time-point. We clarified the method in the revised manuscript to avoid any confusion.

6. Previous studies have indicated that measuring seizure thresholds shifts during the refractory period between bang-induced seizures is a more robust measure of the effects of genotype on seizure sensitivity (e.g., Kuebler and Tanouye, J Neurophys 2000).

We confirmed the seizure thresholds shifts in *Indy* mutants during the refractory period (S1B Fig), as observed in other bang-sensitive mutants (https://pubmed.ncbi.nlm.nih.gov/10669511/).

7. In my opinion, the most important, and somewhat surprising data presented here is included in the section titled “A pair of glutamatergic neurons mediates Indy-dependent seizure suppression”. However, as written, this section is very confusing, and the rational for the testing the hypothesis that Indy is particularly important in “LK” neurons, and what might be the relationship between these neuropeptidergic neurons and the glutamatergic system is not explained or justified. Furthermore, the rationale for the genetic scheme used, how the various tools used specifically address the key questions, and how the approach could help separating developmental versus physiological effects of Indy knockdown, are impossible to follow.

We revised our text to better describe our rationale for experimental design and interpret our results in more detail.

8. The detailed info about the ROGDI gene in the introduction is not really relevant to the main focus of the current paper.

As the other reviewer raised a similar concern (reviewer #2, minor concern #1), we appropriately revised our text for *rogdi*.
