# Supplementary material for: Metabolic flux from the Krebs cycle to glutamate transmission tunes a neural brake on seizure onset
Source: PLoS Genet. 2021 Oct 29;17(10):e1009871. doi: 10.1371/journal.pgen.1009871 (PMC8555787; doi:10.1371/journal.pgen.1009871)
Supplement: Attachment — Submitted filename: ResponseToReviewerComments - 2ndRev.docx [file pgen.1009871.s017.docx]

Please find our point-by-point responses below in blue.

Reviewer #1: The authors have conducted additional experiments, provided new data, and revised the text appropriately in response to most of the comments made by reviewers. Although it is not clear why the seizure-suppressing activity of LHLK neurons are particularly sensitive to reduced glutamatergic transmission, and why their dysfunction induces seizure-like behavior, this study has set the foundation for future investigation of a novel neural circuit governing a bang-sensitive phenotype as well as potential genetic interactions between Indy and other bang-sensitive mutants.

“Seizure-like behaviors, including wing-flapping and abdomen-twitching, were scored in individual flies throughout the recorded video clip”. It’s still not clear to me exactly how seizures are defined. The fly in the video clip displays neither wing-flapping nor abdomen-twitching. Initial paralysis, loss of posture, and uncoordinated leg movements are also considered “seizure”? Please explain.

BSS-positive flies were defined if they displayed initial paralysis upon mechanical stimulus, followed by uncoordinated movements such as wing-flapping, abdominal contractions, or leg-twitching before their recovery of a normal posture. We revised the text to clarify it.

Reviewer #2: The authors have done a good job of addressing my concerns. I have no remaining concerns and think this will be a nice addition to PLoS-Genetics.

Your comments were very constructive to improve the quality of our manuscript.

Reviewer #3: The revised manuscripts is significantly improved. One possibly minor issue that was not addressed in the revision relates to the specific Indy UAS-RNAi line used and it's possible off-target effects. This is particularly important because the transgenic line used in this study is no longer available from public sources, which potentially would make it difficult to replicate the studies by others.

We tested multiple *Indy* RNAi lines (S2C Fig; UAS-*Indy*^RNAi^ #1~#4) and KTS patient-specific allele (Fig 1C, Fig 5B, Fig 5D, S11C Fig, and S11D Fig; UAS-*Indy*^T245M^) to validate *Indy* effects on BSS. Nonetheless, the possible off-target effects of the specific *Indy* RNAi transgene (UAS-*Indy*^RNAi^ #1) were not completely excluded in some experiments (e.g., live-brain imaging), and thus we revised our text in Discussion to indicate the reviewer concern above.
